# Non-clinical development of CER-001

**DOI:** 10.3389/fphar.2015.00220

**Published:** 2015-10-06

**Authors:** Ronald Barbaras

**Affiliations:** Cerenis Therapeutics, Labege, France

**Keywords:** HDL, cholesterol, atherosclerosis, cardiovascular diseases, apolipoprotein A-I

## Abstract

Cardiovascular disease remains the most pressing healthcare issue for the developed world and is becoming so for developing countries. There are no currently approved therapies that can rapidly reduce the burden of unstable, inflamed plaque in the overall coronary vascular bed. High-density lipoprotein (HDL) has multiple actions that could lead to plaque stabilization, such as rapid removal of large quantities of cholesterol from the vasculature through the process of reverse lipid transport, improvement in endothelial function, protection against oxidative damage, and reduction in inflammation. Short-term infusion of HDL-mimetics in animal models as well as in humans has shown promising effects on the plaque size and morphology. Cerenis Therapeutics has developed CER-001, a negatively charged lipoprotein complex consisting of phospholipid and recombinant human apoA-I that mimics the structure and function of natural HDL. Three clinical trials using CER-001 infusions have demonstrated improvements in the carotid wall thickness of patients with familial hypercholesterolaemia and in patients with hypo-alphalipoproteinaemia, as well as an impact on coronary plaque burden measured by intravascular ultrasonography at the lowest tested dose (3 mg/kg) in post-ACS patients. Here, we reviewed the non-clinical data leading to the demonstration that CER-001 is a full HDL mimetic.

## Introduction

Cardiovascular disease remains the most pressing healthcare issue for the developed world and is becoming so for developing countries. A number of chronic therapies are available for long-term management of risk. Short-term therapeutic interventions in subjects with an acute event, such as an episode of acute coronary syndrome (ACS; myocardial infarction or unstable angina), are centered around attempts at reperfusion of the affected myocardium and reduction of thrombus (Piccolo et al., 2015); however, most subsequent events are caused by plaque rupture at a different site. Long-term treatment focuses on reducing cardiovascular risk on top of conventional therapies such as antithrombotic agents, beta-blockers, therapies addressing risk factors, etc, by lowering low-density lipoprotein cholesterol (LDL-C; Hirsh et al., 2015). This strategy has resulted in an impressive reduction in recurrent events of about one third, but still leaves a residual risk (the remaining two thirds of events) unaddressed. There are no currently approved therapies that can rapidly reduce the burden of unstable, inflamed plaque in the overall coronary vascular bed. High-density lipoprotein (HDL) has multiple actions that could lead to plaque stabilization, such as rapid removal of large quantities of cholesterol from the vasculature through the process of reverse lipid transport (RLT), improvement in endothelial function, protection against oxidative damage and reduction in inflammation. In the first randomized trial of HDL infusion in humans, the intravenous administration of recombinant apolipoprotein (apo) A-I_*Milano*_ (a naturally occurring variant of human apoA-I with Arg-173 to Cys substitution) in a complex with phospholipid (ETC-216) produced a significant reduction in coronary atherosclerosis as measured by intravascular ultrasonography (IVUS; Nissen et al., 2003). Another study, using purified apoA-I from human plasma combined with soybean phosphatidylcholine (CSL-111), resulted in significant improvements in the plaque characterization index and coronary score on quantitative coronary angiography in humans (Tardif et al., 2007). This observation, was further confirmed on a trial where atherectomy to excise plaque was performed on superficial femoral artery of patients (*n* = 10) with claudication. After one infusion at 80 mg/kg significant improvement of the lipid content, the macrophage size and the inflammation markers of the plaques were measured (Shaw et al., 2008).

Cerenis Therapeutics has developed CER-001, a negatively charged lipoprotein complex consisting of phospholipid and recombinant human apoA-I that mimics the structure and function of natural HDL. Three clinical trials using CER-001 infusions have demonstrated improvements in the carotid wall thickness of patients with familial hypercholesterolaemia (Hovingh et al., 2015) and in patients with hypo-alphalipoproteinaemia (Kootte et al., 2015), as well as an impact on coronary plaque burden measured by IVUS at the lowest tested dose (3 mg/kg) in post-ACS patients (Andrews et al., 2014; Tardif et al., 2014).

The anti-atherosclerotic properties of HDL infusion were first demonstrated in animals by Badimon et al. (1989) who reported decreased progression of atherosclerotic lesions with weekly infusions of a HDL–very high-density lipoprotein (VHDL) fraction in cholesterol-fed rabbits. Indeed, lipid-rich lesions covered 37.9 ± 6.0% of the intimal aortic surface in the control group (normal saline) but only 14.9 ± 2.1% in the treated group (50 mg/kg of homologous HDL–VHDL protein). Total cholesterol, unesterified cholesterol, esterified cholesterol and phospholipids deposited within the vessel wall were significantly lower in the aortas of the HDL–VHDL-treated group than in the control group, whereas plasma lipid levels were similar in the two groups.

The effects of homologous plasma HDL–VHDL infusions on established atherosclerotic lesions were further evaluated in a separate study with cholesterol-fed rabbits (Badimon et al., 1990). Atherosclerosis was induced by feeding the animals a 0.5% cholesterol-rich diet for 60 days (group 1) or 90 days (group 2), and this was assessed by histological evaluation. A third group received the same diet plus infusions of 50 mg of HDL–VHDL protein per week (isolated from normolipaemic rabbit plasma). At the completion of the study, aortic atherosclerotic involvement and lipid deposition were significantly reduced in the HDL–VHDL-treated animals at 90 days as compared with the 90-day controls (group 2) and the 60-day controls (group 1). This study was the first *in vivo* prospective evidence of the anti-atherogenic effect of HDL–VHDL against pre-existing atherosclerosis, supporting the hypothesis that HDL administration may not only slow lesion progression but may also reduce established atherosclerotic lesions.

The effects of intravenous injection of purified rabbit lipid-free apoA-I on the progression of aortic atherosclerosis in cholesterol-fed rabbits were examined by Miyazaki et al. (1995). Firstly, rabbits were fed a 0.5% cholesterol-rich diet for 90 days. During the last 30 days, the rabbits in the treatment group received weekly infusions of 40 mg of apoA-I. At the completion of the study, the fatty streak lesions in the treated group (23.9 ± 15.6% of total aorta) were significantly decreased compared with those in the untreated group (46.0% ± 24.9%; *P* < 0.05). In a second experiment, cholesterol-fed rabbits (105 days of diet) were maintained for an additional 60 days on a normal diet, during which they received either apoA-I 1 mg every other day or apoA-I 40 mg every week. Although this study did not observe atherosclerosis regression, it did provide the first evidence of the anti-atherogenic effect of homologous apoA-I on the progression of atherosclerosis in cholesterol-fed rabbits.

The effect of recombinant apoA-I_*Milano*_ on intimal thickening after balloon injury in cholesterol-fed rabbits was reported by Ameli et al. (1994). Rabbits received intravenous injections of 40 mg of apoA-I_*Milano*_ as part of a phospholipid complex on alternate days, beginning 5 days before, and continuing for 5 days after, balloon injury of femoral and iliac arteries. ApoA-I_*Milano*_ significantly reduced intimal thickening and macrophage content further implying the therapeutic potential of such a treatment strategy. Later studies, using adenovirus coding for apoA-I, further supported the anti-atherogenic properties of apoA-I, which associates with phospholipids to form nascent HDL (Feng et al., 2009). Several groups have demonstrated that apoA-I complexed with phospholipids behaves like natural HDL after intravenous administration in humans (Nanjee et al., 1999; Nieuwdorp et al., 2008; Feng et al., 2009; van Leuven et al., 2009).

High-density lipoprotein comprises particles of various sizes, depending on the quantity of cholesterol each particle has mobilized and is carrying for transport to the liver for elimination. Newly formed HDL particles (also called pre-β HDL) are the smallest lipid-poor particles; they are essentially “empty” and have the greatest ability to mobilize cholesterol. A natural pre-β HDL is a lipoprotein consisting of apoA-I and phospholipids interacting together to form a small discoidal particle. In a normal individual, pre-β HDL particles represent only 10% of the total cholesterol associated with HDL. These small particles increase in size as they accumulate cholesterol, creating larger, “full,” and mature alpha-HDL particles capable of delivering the acquired cholesterol to the liver for elimination.

Preclinical studies have demonstrated that CER-001, a bioengineered pre-β HDL nanoparticle, has all the biological properties of natural HDL including the capacity to regress atherosclerotic plaque. CER-001 affects all steps of the RLT pathway, as does natural HDL, thus validating the design, functionality and assembly of the particle during the manufacturing process.

• In cellular models, CER-001 was shown to be an effective acceptor of cholesterol.
• *In vivo*, CER-001 mobilizes cholesterol (in a dose-dependent manner) as shown by an increase in HDL-C.
• CER-001 stimulates lecithin cholesterol acyltransferase (LCAT) activity resulting in increased esterification of cholesterol.
• CER-001 increases elimination of cholesterol in the feces.
• Finally, CER-001 specifically mimics the behavior of a natural pre-β HDL as shown by the similar dose-dependency of atherosclerosis regression for CER-001 and a natural pre-β HDL.

Among the different HDL properties, the capacity to remove cholesterol from cholesterol-loaded macrophages present in the atherosclerotic plaques could be a pre-requisite for defining functional HDL particles.

## CER-001 Composition; Effect of the Charge

**FIGURE 1:**
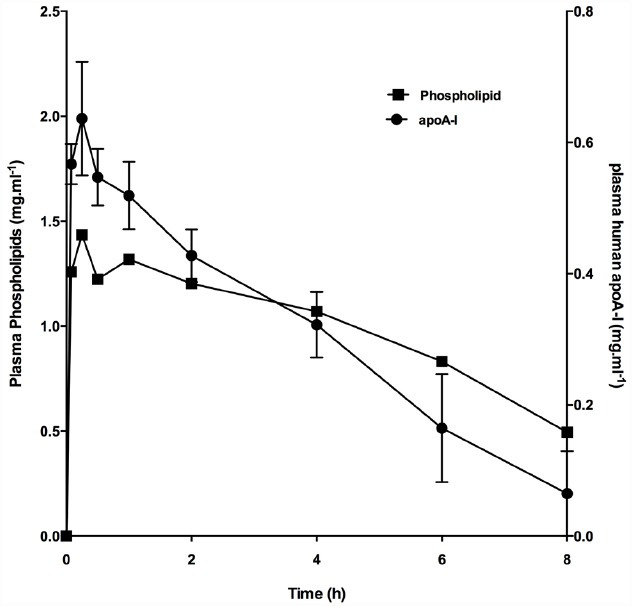
**Increase in plasma phospholipid and apoA-I following infusion of CER-001.** CER-001 (20 mg/kg) was infused into fasted rabbits. There were three animals per group. At various times post dose, plasma phospholipid and apoA-I levels were measured. Plasma human apoA-I levels were measured by a human apoAI-specific enzyme-linked immunosorbent assay (ELISA). Baseline values 1.17 g/l were subtracted to determine the increase in plasma phospholipid levels. The values had returned to baseline levels by 30–34 h post dose.

The phospholipid species used in CER-001 were selected for their ability to form a discoidal complex with a strong affinity for cholesterol (Mattjus and Slotte, 1996; Ramstedt and Slotte, 1999), a negative charge to slow clearance by the kidney (Guasch et al., 1993), their ability to trigger recognition by the liver (Guasch et al., 1993; Rigotti et al., 1995) and also for the high stability of the complexes in the circulation (Figure 1; Baron et al., 2010). Early studies focused on optimizing the composition to produce a homogeneous population of stable protein/lipid complexes that eluted in the same fraction as natural pre-β HDL in column chromatography (size) and migrated similarly to natural HDL in gel electrophoresis (charge; Tardy et al., 2015). The formulation for CER-001 consists of a recombinant human apoA-I produced by genetically modified Chinese hamster ovary (CHO) cells and a protein-to-lipid weight ratio of 1:2.7. The phospholipid component consists of natural egg sphingomyelin and dipalmitoylphosphatidylglycerol in a 97:3 weight ratio (Tardy et al., 2015).

## CER-001 and Cholesterol Efflux

Determination of cholesterol efflux from macrophages is considered the hallmark of HDL activity/capacity for recycling cholesterol from cells (Khera et al., 2011). Cholesterol is a membrane constituent that maintains structural domains important in the regulation of vesicular trafficking and signal transduction. In most cells, cholesterol is not catabolized. Thus, the regulation of cellular sterol efflux plays a crucial role in cellular sterol homeostasis. Cellular sterol can efflux to extracellular sterol acceptors by non-regulated, passive diffusion mechanisms as well as by an active, regulated, energy-dependent process mediated by the adenosine triphosphate-binding cassette (ABC) A1 (ABCA1) transporter and, to a lesser extent, the ABCG1 transporter. The ABCA1 transporter mediates the cellular efflux of lipids, in particular phospholipids and cholesterol, from peripheral tissues to lipid-poor apoA-I in the plasma to form pre-β HDL particles. In preclinical models, the role of both hepatic and extrahepatic ABCA1 in the assembly of the HDL particle has been demonstrated. In humans, homozygous mutations in the ABCA1 gene leading to defective or non-functional ABCA1 receptors result in Tangier disease, characterized by profoundly decreased HDL-C, apoA-I and apoA-II levels, reduced total cholesterol, LDL-C, and apoB, elevated plasma triglyceride levels leading to an increase in cardio-vascular disease. With ABCA1 deficiency, apoA-I is rapidly cleared before it is able to acquire cholesterol; therefore, the cholesterol storage disorder that occurs with ABCA1 mutations may be more a consequence of HDL deficiency than a direct consequence of dysfunctional ABCA1 (Attie et al., 2001).

It was previously shown that CER-001 stimulated the cholesterol efflux from mouse macrophages in a similar way to HDL_3_. This similarity was also noted when evaluated in human macrophages (THP-1), demonstrating CER-001 to be a functional HDL particle that captures cholesterol from macrophages in different species (Tardy et al., 2014). The CER-001-mediated cholesterol efflux increased in a dose-related manner with concentrations of the complex and was similar to the HDL_3_-mediated cholesterol efflux. The HDL_3_ cholesterol efflux determined in the experiment is similar to previous reports in the literature (Cogny et al., 1996; Bortnick et al., 2000; Suc et al., 2003). CER-001 is fourfold more potent than lipid-free apoA-I in effluxing cholesterol from macrophages. In these defined experimental conditions, cholesterol efflux from J774 macrophages reached a plateau at 100 μg/ml of CER-001 (Tardy et al., 2014). These data suggest that the observed cholesterol efflux was specific for the acceptor added on the cells.

Up-regulation of ABCA1 expression by cyclic adenosine monophosphate (cAMP) increases the cholesterol efflux in macrophages (named ABCA1-specific efflux; Oram et al., 2000). Under these conditions, a high lipid-free apoA-I cholesterol efflux was observed as described previously (Oram et al., 2000). This lipid-free apoA-I efflux is higher than CER-001 and HDL_3_ cholesterol efflux (Tardy et al., 2015). The number of cholesterol acceptors (i.e., the HDL_3_, CER-001 and delipidated apoA-I) in the culture medium of the cell (25 μg/ml) is high enough to accommodate all the cellular cholesterol and so is not a limiting factor that could explain this observation. One hypothesis is that a down-regulation of the ABCA1 by HDL_3_ and CER-001 would impair the cholesterol efflux capacity of the macrophages. Thus, an ABCA1 mRNA down-regulation induced by CER-001 and HDL_3_ could be associated with a reduction in the ABCA1-specific cholesterol efflux from macrophages. Indeed, it was observed that at CER-001 or HDL_3_ concentrations >20 μg/ml, ABCA1 mRNA expression decreases to half that of control values in J774 macrophages. Delipidated apoA-I did not change the ABCA1 mRNA level even at concentrations up to 300 μg/ml (Tardy et al., 2015).

ABCA1 protein content was measured in macrophages treated with CER-001, HDL_3_, or apoA-I (Oram et al., 2000). As expected, the addition of cAMP boosted the ABCA1 protein level compared with untreated macrophages. In the presence of a high dose (250 μg/ml) of CER-001 or HDL_3_, the ABCA1 protein level in the membrane was halved. Delipidated apoA-I did not affect macrophage ABCA1 protein content (Tardy et al., 2015).

In summary, ABCA1 mRNA and protein level are decreased after high dose treatment with CER-001 or HDL_3_. Further analysis is needed, but one can hypothesize that the down-regulation of ABCA1 could occur when a low level of cholesterol is reached in the macrophages. Indeed, cholesterol efflux from macrophages measured in the presence of β-cyclodextrin also down-regulates ABCA1 expression. β-cyclodextrin is a well-known potent acceptor of cholesterol, independent of any cellular transporters and/or receptors (Field et al., 2008).

## CER-001 and *In Vitro* Inflammation

Chronic inflammation is considered another hallmark of the development and progression of the atherosclerotic plaque (Ross, 1999). Lipopolysaccharide (LPS) and tumor necrosis factor-α (TNF-α) are implicated in the induction of cytokines and adhesion molecule secretion. These mediators are responsible for increasing monocyte infiltration into the arterial wall, promoting the development of atherosclerotic plaques. To test whether CER-001 regulated the expression of LPS- or TNF-α-induced inflammatory molecules such as interleukin IL-6, IL-8, monocyte chemoattractant protein-1 (MCP-1) and granulocyte-macrophage colony-stimulating factor (GM-CSF), the media of human umbilical vein endothelial cells (HUVEC) exposed to LPS in the absence or presence of CER-001, HDL_2_, HDL_3_ or lipid-free apoA-I, was collected (Tardy et al., 2014). Increasing concentrations of CER-001 reduced the secretion of all the cytokines tested in a dose-dependent manner starting at 5 μg/ml, with complete inhibition at 500 μg/ml. Treatment with purified HDL_2_ and HDL_3_ lipoproteins, and lipid-free apoA-I (500 μg/ml) had a modest effect on cytokine secretions. When LPS was added to HUVEC, the cells secreted inflammatory molecules, which in turn were able to recruit non-adherent monocytes. In the presence of LPS, a dose-dependent decrease in THP1 monocyte recruitment was observed when CER-001 was added, with a significant decrease starting at 5 μg/ml. This observation was further confirmed by using TNF-α as an alternative inducer of the inflammatory mediators.

## CER-001 and Cholesterol Mobilization *In Vivo*

**FIGURE 2:**
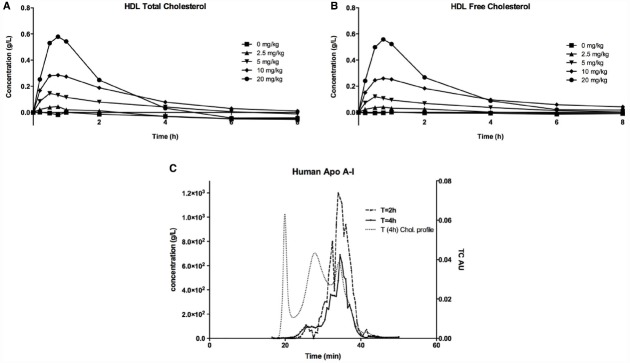
**Increase in plasma HDL (A) total cholesterol and HDL (B) unesterified cholesterol following infusion of CER-001.** CER-001 was infused into fasted rabbits at doses of: 0 (squares), 2.5 (triangles), 5 (inverted triangles), 10 (diamonds), or 20 (circles) mg/kg. There were three animals per group. At various times post dose, plasma HDL total and unesterified cholesterol were measured. Baseline values were subtracted to determine the increase in plasma HDL cholesterol levels. Baseline HDL total and unesterified cholesterol ranged from 0.19 to 0.45 g/l for total and from 0.06 to 0.13 g/l for unesterified cholesterol. By 30–34 h post dose the values had returned to a value at or below baseline. **(C)** ApoA-I HPLC gel permeation chromatography profile. This is an example of an elution profile for human apoA-I at 2 h (dashed line) and 4 h (plain line) after infusion of CER-001 (20 mg/kg). Light dashed lines correspond to total cholesterol profile at 4 h. The elution times of 30 to 40 min correspond to the HDL fraction, while 18–22 min and 25–30 min correspond to VLDL and LDL, respectively.

**FIGURE 3:**
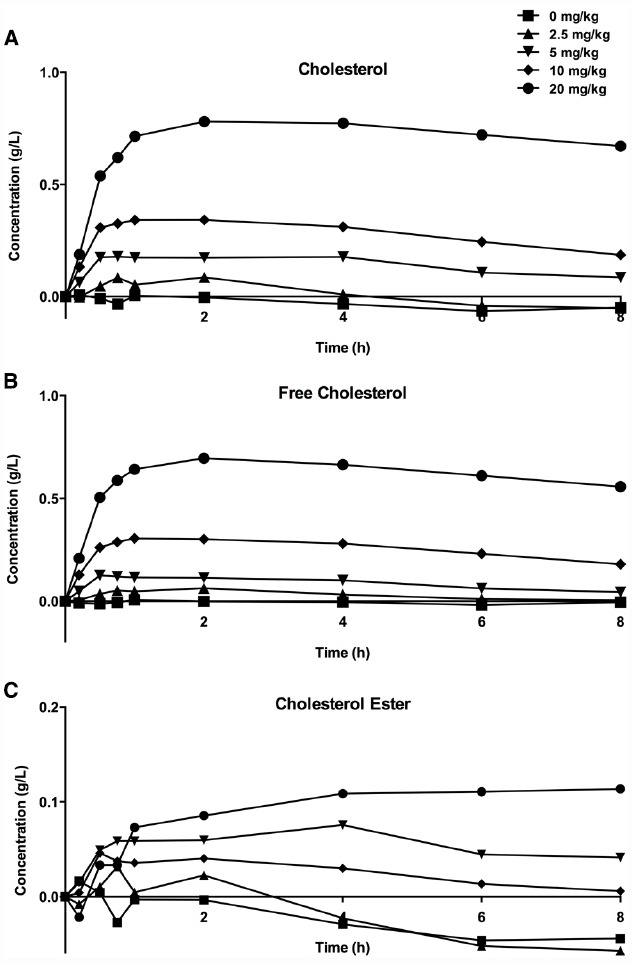
**Increase in plasma total (A), unesterified (B), and esterified (C) cholesterol levels following infusion of CER-001.** CER-001 was infused into fasted rabbits at doses of: 0 (squares), 2.5 (triangles), 5 (inverted triangles), 10 (diamonds) or 20 (circles) mg/kg. There were three animals per group. At various times post dose, plasma total, unesterified and esterified cholesterol levels were measured. Baseline values were subtracted to determine the increase in cholesterol levels. The baseline values ranged from 0.41 to 1.08 g/l for total cholesterol, from 0.13 to 0.3 g/l for unesterified cholesterol, and from 0.28 to 0.78 g/l for esterified cholesterol. By 30–34 h post dose the values had returned to a value at or below baseline.

When CER-001 was infused into rabbits at doses ranging from 2.5 to 20 mg/kg, a dose-dependent increase in plasma phospholipids and human apoA-I was observed, reflecting the infusion of the apoA-I and phospholipid content of CER-001. The prolonged residence of apoA-I in the HDL fraction suggests that CER-001 is stable throughout the cholesterol mobilization process (Figure 3C). Dose-dependent cholesterol mobilization was evident by the increase in plasma unesterified cholesterol as well as esterified cholesterol (Figure 2). The increase of esterified cholesterol could be the consequence of LCAT activity, the enzyme responsible for the cholesterol esterification in plasma. HDL-C (unesterified and esterified cholesterol) increased at doses of 5 mg/kg and greater, with peak increases in HDL total cholesterol occurring 30 min after initiation of the CER-001 infusion (Figure 3). The plasma phospholipid and cholesterol values returned to baseline levels by 30 to 34 h post dose (Baron et al., 2010).

The cholesterol mobilization profile following a single dose of CER-001 (10 mg/kg) in C57Bl/6J mice was similar to that observed in rabbits. Following injection, the human apoA-I reached maximal concentration of over 300 μg/ml in 15 min in the mouse plasma. After 4 h, half of the injected complex had been eliminated, and 15% of human apoA-I was detected in the plasma of the injected mice. The plasma total cholesterol curve after single-dose CER-001 treatment paralleled the apoA-I pharmacokinetic curve (i.e., they were superimposable), reaching a maximum at about 30 min. Again, half of this pool of cholesterol was cleared from plasma in the next 4 h. As expected, most of the plasma cholesterol mobilized was unesterified cholesterol (Tardy et al., 2014).

## CER-001 Increases Plasma Cholesterol Clearance of LDLr^–/–^ Mice

Reverse lipid transport is the process by which excess cellular cholesterol is liberated from peripheral tissues by HDL and delivered to the liver for elimination via the intestine (Lewis and Rader, 2005). LDL receptor knockout (LDLr^–/–^) mice (an animal model of familial hypercholesterolaemia) were fed with a high fat diet (HFD) for 10–11 weeks to develop atherosclerotic lesions. After this time, CER-001 (10 mg/kg) was given intravenously every second day starting either at week 8 (10 doses) or at week 9 (5 doses). The lipid analysis of the livers showed that unesterified cholesterol and cholesterol ester concentrations were increased by 10% in the livers of mice injected with CER-001 (compared with control mice). In addition, the cholesterol excretion in feces was also increased (by 27%) in mice treated with CER-001 at a dose of 10 mg/kg. In conclusion, infusion of CER-001 increased cholesterol clearance by the liver into the feces primarily in the form of unesterified cholesterol, thus demonstrating that CER-001 performed the final step of RLT in these mice (Tardy et al., 2014).

## CER-001 Inhibits Plaque Progression and Plaque Inflammation in LDLr^–/–^ Mice

LDLr^–/–^ mice, following 10–11 weeks on a HFD, developed atherosclerotic lesions in the aortic root, characterized by fatty streaks and accumulation of macrophage foam cells (Tardy et al., 2014). CER-001 (10 mg/kg) given intravenously every second day starting at either week 8 (10 doses) or at week 9 (5 doses) reduced plaque size by 17% (5 doses) and 32% (10 doses), and lipid content by 17% (5 doses) and 23% (10 doses), respectively. Plaque inflammation was also reduced; there was an 80% decrease of lesion macrophage content (5 doses), and vascular cell adhesion molecule-1 (VCAM-1) expression decreased by 16% (5 doses) and 22% (10 doses). These data demonstrate that infusion of CER-001 in LDLr^–/–^ mice slowed the formation of atherosclerotic plaques and reduced the associated inflammation after short-term multiple-dose treatment of CER-001 (Tardy et al., 2014).

## Attenuated Inhibition of Atherosclerotic Plaque Progression in apoE^–/–^ Mice at High HDL Doses is Associated with Down-Regulation of ABCA1

The HDL mimetic, CER-001, as well as purified HDL, induce down-regulation of ABCA1 in a dose-dependent manner. This observation needs to be considered in the context of previous observations. Historically, ABCA1 in macrophages was observed to be down-regulated *in vitro* by incubation with HDL (Langmann et al., 1999). *In vivo*, a scrutiny analysis of the first randomized trial of HDL infusion in humans using an apoA-I_*Milano*_/phospholipid complex has shown a trend for less efficacy at the higher dose of 45 mg/kg than at the lower dose of 15 mg/kg (Nissen et al., 2003). A similar observation was made during the CER-001 clinical trial (CHI-SQUARE) where a promising effect on plaque regression was detected only at the lowest dose of 3 mg/kg (Tardif et al., 2014), with an attenuation of effect at the higher doses.

We examined the CER-001 and HDL_3_ dose response *in vivo* by assessing plaque formation in ligatured carotid of apoE^–/–^ mice fed a HCD. The left carotid of apoE^–/–^ mice was ligatured, and groups of mice were subsequently fed with a HCD and treated (retro-orbital injection) every second day with different doses of CER-001 or HDL_3_ for two weeks. After eight infusions, a gradually greater decrease of unesterified and total cholesterol content in ligatured carotids was observed as the dose of CER-001 or HDL_3_ increased from 2 to 5 to 10 mg/kg. No decrease in cholesterol content was observed for dose levels >10 mg/kg; the level of cholesterol content above this dose was the same as that observed in untreated mice (Tardy et al., 2015). The ABCA1 protein content in the ligatured carotids inversely followed that of cholesterol content in a similar dose-related manner. At doses at which plaque formation in carotid was maximally inhibited (5 mg/kg of CER-001 and HDL_3_), carotid artery ABCA1 protein levels were similar to those of untreated animals. In contrast, at the 50 mg/kg dose of CER-001 or HDL_3_, the carotid cholesterol content of plaque burden was similar to that of untreated animals, and the carotid artery ABCA1 protein level was decreased by half compared with untreated animals. Thus, down-regulation of macrophage ABCA1 induced by elevated concentrations of CER-001 or HDL_3_ appears to attenuate the efficacy of the infused HDL to inhibit plaque formation in the apoE^–/–^ mice ligatured carotid model; however, at lower doses, increasing the dose of CER-001 and HDL_3_ up to that observed threshold slowed progression of carotid plaque while maintaining normal levels of ABCA1 expression.

## As a Matter of Conclusion

ABCA1 mRNA and protein level are decreased after high dose treatment with CER-001 or HDL_3_. Further analysis is required, but one may hypothesize that the down-regulation of ABCA1 could occur when a low level of cholesterol is reached in the macrophages. Another ABC transporter, ABCG1, also seems to be involved in the cellular cholesterol efflux process mediated by HDL particles. ABCG1 seems to act in addition to the action of ABCA1 on cholesterol efflux with an appetency for larger HDL particles than ABCA1 (Tarling, 2013). Unfortunately, in humans, the real effect of ABCG1 on global cholesterol efflux and on the development of the atherosclerotic plaque burden has not been evaluated. Further study of a potential down-regulation of ABCG1 by HDL, identical (or different) to ABCA1 could be very informative for further understanding general cellular cholesterol homeostasis.

We suggest that in order to have a full and efficient action of HDL or HDL-mimetics on cholesterol efflux in macrophages, we need to find the right dose to avoid down-regulation of ABCA1, the “gate-keeper” of cholesterol efflux from the cell. This would allow a continued cholesterol efflux toward the HDL acceptors and consequently would facilitate a decrease in atherosclerotic plaque burden. Further clinical trials with HDL mimetics and CETP inhibitors, which increase the HDL-C plasma concentration, need to take into account this phenomenon of dose-dependent HDL-mediated down-regulation of ABCA1.

### Conflict of Interest Statement

The author declares that the research was conducted in the absence of any commercial or financial relationships that could be construed as a potential conflict of interest.
